# Bevacizumab plus FOLFIRI or FOLFOX in chemotherapy-refractory patients with metastatic colorectal cancer: a retrospective study

**DOI:** 10.1186/1471-2407-9-347

**Published:** 2009-09-28

**Authors:** Astrid Lièvre, Emmanuelle Samalin, Emmanuel Mitry, Eric Assenat, Christine Boyer-Gestin, Céline Lepère, Jean-Baptiste Bachet, Fabienne Portales, Jean-Nicolas Vaillant, Marc Ychou, Philippe Rougier

**Affiliations:** 1 Gastroenterology and Digestive Oncology Unit, Assistance Publique Hôpitaux de Paris, Hôpital Ambroise Paré, Boulogne Billancourt, France; 2 EA4340, Université Versailles Saint-Quentin, UFR de médecine Paris Ile de France Ouest, Saint-Quentin en Yvelines, France; 3 Digestive Oncology Unit, Centre Régional de Lutte contre le Cancer Val d'Aurelle - Paul Lamarque, Montpellier, France

## Abstract

**Background:**

The anti-VEGF antibody bevacizumab associated with an irinotecan or oxaliplatin-based chemotherapy was proved to be superior to the chemotherapy alone in first or second line treatment of metastatic colorectal cancer (mCRC). However, it was reported to have no efficacy in 3^rd ^or later-line, alone or with 5FU. The aim of this study was to evaluate the activity of bevacizumab combined with FOLFIRI or FOLFOX in mCRC who have failed prior chemotherapy with fluoropyrimidine plus irinotecan and/or oxaliplatin.

**Methods:**

Thirty one consecutive patients treated between May 2005 and October 2006 were included in this retrospective study. All of them have progressed under a chemotherapy with fluoropyrimidine plus irinotecan and/or oxaliplatin and received bevacizumab (5 mg/kg) in combination with FOLFIRI or simplified FOLFOX4 every 14 days.

**Results:**

Ten patients (32.2%) had an objective response (1 CR, 9 PR) and 12 (38.8%) were stabilized. The response and disease control rates were 45.4% and 100% when bevacizumab was administered in 2^nd ^or 3^rd ^line and 25% and 55% in 4^th ^or later line respectively (p = 0.024 and p = 0.008). Among the patients who had previously received the same chemotherapy than that associated with bevacizumab (n = 28) the overall response rate was 35.7% and 39.3% were stabilized. Median progression free survival (PFS) and overall survival (OS) were of 9.7 and 18.4 months respectively. Except a patient who presented a hypertension associated reversible posterior leukoencephalopathy syndrome, tolerance of bevacizumab was acceptable. A rectal bleeding occurred in one patient, an epistaxis in five. Grade 1/2 hypertension occurred in five patients.

**Conclusion:**

This study suggests that bevacizumab combined with FOLFOX or FOLFIRI may have the possibility to be active in chemorefractory and selected mCRC patients who did not receive it previously.

## Background

Colorectal cancer (CRC) is one of the most common human malignancies with more than 300,000 cases both in the United States and in the European Union each year. In the past decade, significant improvements have been performed in response rates, progression-free survival (PFS) and overall survival (OS) [1-4]. Despite these improvements, mainly due to the development of new combinations of standard chemotherapy including 5 fluorouracil, irinotecan and oxaliplatin, nearly all patients with metastatic CRC (mCRC) will die from their disease. Recently, new therapeutic agents targeting molecular events involved in colorectal carcinogenesis have been developed, including bevacizumab, a recombinant humanized monoclonal antibody, which binds to the vascular endothelial growth factor (VEGF) with a high specificity and prevents its interaction with receptors on endothelial cells. VEGF plays a key role in angiogenesis, which is involved in the development of carcinogenesis, tumor growth and malignant dissemination. Therefore, bevacizumab inhibits the activation of VEGF-receptor-mediated signaling pathways and resultant biological effects [5]. This antiangiogenic agent, added to a 5-fluorouracil (5FU) ± irinotecan-based chemotherapy as first-line treatment, has been shown to improve response rates and survival of mCRC patients when compared to the chemotherapy alone [6-8]. An improvement of PFS was also shown in first-line with the addition of bevacizumab to oxaliplatin-based chemotherapy [9]. A randomized phase III study also reported a clinical efficacy of the association of bevacizumab and FOLFOX4 as second-line in mCRC patients previously treated with a fluoropyrimidine and irinotecan, with a significant improvement in response rates, PFS and OS when compared to FOLFOX4 alone [10].

In Europe, bevacizumab was approved by the EMEA (European Medicines Evaluation Agency) in the beginning of 2005 and many CRC patients could not have received it in first or second-line treatment before this date. Moreover, it was reported to have no efficacy in 3rd or later-line, alone or with 5FU [10,11]. The aim of this retrospective study was to evaluate the activity of bevacizumab combined with a chemotherapy with 5FU/LV and irinotecan or oxaliplatin in mCRC who have failed prior chemotherapy with fluoropyrimidine plus irinotecan and/or oxaliplatin.

## Methods

### Patient characteristics

In this retrospective study, we included all the patients prospectively registered in two centers (Hôpital Ambroise Paré, Boulogne Billancourt and Centre Régional de Lutte contre le Cancer Val d'Aurelle, Montpellier, France) with histologically proven mCRC who had been previously treated with a fluoropyrimidine (e.g., fluorouracil or capecticabine) plus irinotecan and/or oxaliplatin with no response to treatment (as defined by tumor progression according to the RECIST criteria [12] or unacceptable toxicity) and who then received bevacizumab in combination with FOLFIRI or simplified FOLFOX4 between May 2005 and October 2006. The decision of treatment was always taken during multidisciplinary staff for patients who had not the opportunity to receive bevacizumab at an earlier line of chemotherapy. The usual exclusion criteria were a history of major surgery within 28 days, a thrombotic or bleeding event within 6 months, a hypertension, a clinically significant cardiovascular disease, a hypertension, a therapeutic anticoagulation and the presence of brain metastases. All medical files of the patients were registered prospectively in a computerised database (after national registry council (CNIL) authorization). The following data were collected and analyzed: age and performance status (according to WHO criteria) at the time of the first cycle of bevacizumab, gender, primary tumor site (colon or rectum), number and localization of metastatic sites, previous anticancer drugs received and tumor response to them. This retrospective study was proposed in January 2007 and approved by the local scientific and ethical committee.

### Treatment protocols

Patients were treated with bevacizumab given at a dose of 5 mg/kg on day 1 every two weeks, followed by a 2-hour infusion of 400 mg/m^2 ^of leucovorin, given simultaneously with a 2-hour infusion of 85 mg/m^2 ^of oxaliplatin, followed by a bolus of 400 mg/m2 of 5FU and then a 46-hour infusion of 2400 mg/m^2 ^of 5FU (simplified FOLFOX4) or the same regimen with a 90 minutes (min) infusion of 180 mg/m^2 ^of irinotecan instead of the infusion of oxaliplatin (FOLFIRI) (1 cycle = 14 days). The first infusion of bevacizumab was given over 90 min, the second over 60 min and the following over 30 min when previous infusions were well tolerated.

### Tumor evaluation

All the patients must have received at least four cycles of chemotherapy with bevacizumab to be evaluable, but two patients had evidence of a clinical progression before the radiologic evaluation of tumor response and received only three cycles of bevacizumab.

Tumour response was prospectively assessed every four cycles according to RECIST criteria by computerized tomography-scan. Treatment was repeated until the occurrence of disease progression or unacceptable toxicity.

Toxicity was assessed according to the Common Toxicity Criteria version 2.0[13].

### Statistical analysis

Fisher's exact test was used to compare response and stabilization rates between groups. The PFS was calculated as the period from the first day of bevacizumab treatment to the date of tumor progression, to death from any cause or to the date of the last follow-up at which data point was censored. The OS time was calculated as the period from the first day of bevacizumab treatment until death of any cause or until the date of the last follow-up, at which data point was censored. Both PFS and OS were estimated by the Kaplan-Meier method and compared using the log-rank test. Analysis was carried out using the STATA software package (College Station, Texas). The level of significance was set at p = 0.05.

## Results

### Patient characteristics

Between May 2005 and October 2006, a total of 31 patients (17 men, 14 women, median age: 60 years) with mCRC resistant to fluoropyrimidine plus irinotecan (96.7%) and/or oxaliplatin (90.3%) received bevacizumab combined with FOLFIRI or FOLFOX4. A total of 373 cycles of bevacizumab combined with chemotherapy were administered, with a median of 12 cycles per patient (range: 3 to 35 cycles).

Patients characteristics are detailed in the table 1. All the patients had a good WHO performance status (≤ 2) at the beginning of bevacizumab therapy except one (WHO PS = 3). The number of metastatic sites were limited to one or two organs in 77% of the cases (24 patients), and metastases were mostly located in the liver (84%) or lung (42%). Twenty seven patients (87%) had previously received fluoropyrimidine, irinotecan and oxaliplatin and 19 (61%) had received cetuximab. In 19 out of 20 patients who received bevacizumab in 4th-line or further (L4+), cetuximab was previously used and failed, five patients previously received a monotherapy of fluoropyrimidine (capecitabine ou 5FU), four patients were treated by the association of capecitabine and mitomycin-C and five by hepatic arterial infusion of oxaliplatin and intraveinous LV5FU2. Bevacizumab was combined with FOLFIRI in 19 cases and with FOLFOX in 12 cases and was administered equally as third, fourth and fifth or later-line (32% in each group). One patient was treated in second line but he was progressive under a combination of 5FU, irinotecan and oxaliplatine (FOLFIRINOX regimen).

**Table 1:** Patients characteristics

Characteristics	
Sex: Male/Female	17/14
Age (years)	
Median	60
Range	24-81
WHO Performance status, n(%)	
0	10 (32)
1	16 (52)
2	4 (13)
3	1 (3)
Primary tumor location, n (%)	
Colon	23
Rectum	8
Metastatic sites, n (%)	
Liver	26 (84)
Lung	13 (42)
Peritoneum	5 (16)
Others	12 (39)
Number of metastatic sites, n (%)	
1	15 (48)
2	10 (32)
≥ 3	7 (23)
Chemotherapy associated with bevacizumab, n (%)	
FOLFIRI	19 (61)
FOLFOX4	12 (39)
Line number of bevacizumab, n (%)	
2^nd ^line	1(3.25)
3rd line	10 (32.25)
4th line	10 (32.25)
5th or later-line	10 (32.25)
Previous chemotherapy	
Fluoropyrimidine + irinotecan + oxaliplatin	27
Fluoropyrimidine + irinotecan	3
Fluoropyrimidine + oxaliplatin	1
Cetuximab	19

### Response rates

The overall response rate was 32.2% (table 2). One patient (3.2%) had a complete response and 9 (29%) a partial response. Among them, one patient could have a resection of liver metastases after a partial response to FOLFIRI plus bevacizumab and was alive without relapse at the last follow-up. Twelve patients (38.8%) had a stable disease, with a disease control rate of 71%. The response rate was 45.4% when bevacizumab was administered in 2nd and 3rd-line (L2-L3) and 25% when it was administered in 4th-line and further (L4+) respectively (table 2; p = 0.024). It was 36.9% when bevacizumab was associated with FOLFIRI and 25% when associated with FOLFOX (p = 0.6). The disease control rate was 100% when bevacizumab was administered in L2-L3 and 55% when it was administered in L4+ respectively (table 2; p = 0.008). Most of the patients (n = 28; 90%) had previously received the same chemotherapy (FOLFOX or FOLFIRI) than that associated with bevacizumab. Among these patients, the overall response rate was 35.7% and 39.3% were stabilized (table 2).

**Table 2:** Overall response rates

	Patients (n)	CR (%)	PR (%)	SD (%)	PD (%)
Overall	31	1 (3.2)	9 (29)	12 (38.8)	9 (29)
2^nd ^and 3^rd ^line	11	0 (0)	5 (45.5)	6 (55.5)	0 (0)
4^th ^and later line	20	1 (5)	4 (20)	6 (30)	9 (45)
FOLFIRI	19	1 (5.3)	6 (31.6)	8 (42.1)	4 (2.1)
FOLFOX	12	0 (0)	3 (25)	4 (33.3)	5 (41.7)
Same CT previously used					
Yes	29	1 (3.5)	9 (31)	11 (37.9)	8 (27.6)
No	2*	0 (0)	0 (0)	1 (50)	1 (50)

CR; complete response, CT; chemotherapy; PR; partial response, SD; stable disease, PD; progression disease. * no previous treatment by FOLFOX in two patients treated by bevacizumab + FOLFOX

### Survival

**Figure 1:**
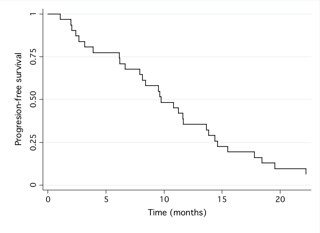
**This figure displays a graph showing 'Progression free survival'**.

**Figure 2:**
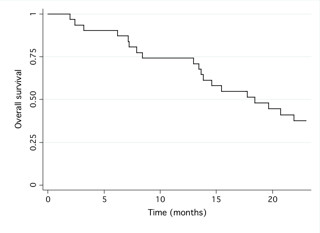
**This figure displays a graph demonstrating 'Overall survival'**.

With a median follow-up period of 35.9 months (range: 33 to 37.4 months) from the beginning of bevacizumab administration, the median PFS was 9.7 months (95% confidence interval (CI): 6.6-13.6) (figure 1) and the median OS was 18.4 months (95%CI: 13.6- not reached) (figure 2).

### Toxicity

There was no toxic death. The incidence of haematological and non-hematological toxicity is summarized in Table 3. A grade 3/4 toxic event occurred in 19,3% of patients. A cytotoxic dose reduction or interruption and a delay in chemotherapy cycles was required in 51% and 13% of patients respectively. As concerns bevacizumab-induced toxicity, only one interruption was required in a patient who presented a reversible posterior leukoencephalopathy syndrome attributed to bevacizumab therapy because of associated hypertension. This syndrome was revealed by a generalized tonic-clonic seizure and diagnosed on magnetic resonance imaging of the brain [14]. Except this severe adverse event, tolerance of bevacizumab was acceptable, without bowel perforation, thromboembolism event, severe bleeding or hypertension. A minimal rectal bleeding occurred in one patient and a grade 1/2 hypertension in five patients, which was easily manageable by an antihypertensive treatment. An epistaxis also occurred in five patients.

**Table 3:** Chemotherapy-induced toxicity (NCI-CTC version 2.0)

	Grade 1, n	Grade 2, n	Grade 3, n	Grade 4, n
Neutropenia	0	3	1	0
Thrombocytopenia	2	1	0	0
Anemia	0	2	1	0
Diarrhea	10	4	1	0
Nausea-vomiting	10	9	2	0
Alopecia	3	0	0	0
Hand-foot syndrome	0	1	0	0
mucositis	6	0	2	0
Neurotoxicity	3	3	4	0
Asthenia	9	7	3	0
Allergic reaction	0	1	0	0
Maximum/patient (%)	4 (12,9)	15 (48,4)	6 (19,3)	0 (0)

NCI-CTC: National Cancer Institute Common Toxicity Criteria.

## Discussion

This study reports an activity of bevacizumab at the dose of 5 mg/kg combined with FOLFOX or FOLFIRI regimen in patients who have failed prior chemotherapy with fluoropyrimidine, irinotecan and/or oxaliplatin. Indeed, 32% of the patients could achieved an objective response and one patient could secondary have a curative resection of its liver metastases. The disease control rate was superior to 70%. The addition of bevacizumab to FOLFIRI or FOLFOX was also associated with a PFS and a OS of 9.7 months and 18.4 months respectively. These response and survival rates are higher than those reported by Giantonio et al. with the combination of FOLFOX plus bevacizumab in second line treatment in patients previously treated by fluoropyrimidine and irinotecan, but this might be certainly explained by the small size and the retrospective nature of our study. Furthermore, patients who received bevacizumab during the period of the study were highly selected because bevacizumab was not registered in this setting but only in first line treatment. For these reasons, our results have to be taken with caution. However, they are nonetheless interesting in third-line treatment where few molecules have been shown to be effective. In patients pretreated with fluroropyrimidine, irinotecan and/or oxaliplatin, several studies have reported poor response and survival rates with standard chemotherapy such as the association of mitomycine C and capecitabine [15-18]. The antibodies against the Epidermal Growth Factor Receptor (EGFR) are the only therapy to have shown their efficacy in chemorefractory CRC patients. Cetuximab was the first to show a significant response rate in association with irinotecan in irinotecan-refractory mCRC in the phase II BOND study, in which PFS and OS were of 4.1 and 8.6 months respectively [19]. The superiority of cetuximab monotherapy over best supportive care (BSC) in terms of survival was demonstrate more recently [20]. The fully human anti-EGFR antibody panitumumab was also associated with a longer PFS when compared to BSC in a recent randomized phase III trial [21]. However, it is now clearly demonstrated that patients with a tumor *KRAS *mutation are resistant to anti-EGFR antibodies and do not benefit from this targeted therapy [22-26]. Bevacizumab should therefore be considered for these patients after failure of conventional chemotherapy. In our study, 61% of the patients had previously received cetuximab and were so resistant to all potentially efficient therapies approved in the treatment of mCRC.

In patients who were previously treated with the same chemotherapy regimen (90% of all patients included), the addition of bevacizumab to this regimen allowed a response rate of 35.7% and a stabilization of 39.3%, which suggests that the antiangiogenic therapy may circumvent resistance to conventional chemotherapy by allowing a more efficient delivery of chemotherapeutic agents. Tumor vasculature is structurally and functionally abnormal, which results in an heterogeneity in tumor blood flow with interstitial hypertension, hypoxia and acidosis [27]. Hypoxia could therefore renders tumor cells resistant to several cytotoxic drugs by interfering with the penetration of these drugs throughout the tumor. The concept of "normalization" of tumor vasculature induced by bevacizumab, developed by Jain [28] who demonstrated with some colleagues this effect of VEGF blockade in rectal carcinoma [29], may therefore explain the circumvention of resistance to conventional therapies observed in our study. This phenomenon do not seem to be observed with the combination of an anti-EGFR antibody with bevacizumab considering the disappointing results of two recent randomized studies in which the addition of cetuximab or panitumumab to bevacizumab in combination with a conventional chemotherapy was not associated with any benefit but on the contrary perhaps with a deleterious effect [30,31], which remains to be elucidated.

A first randomized phase II trial of bevacizumab plus bolus 5FU/LV compared to 5FU/LV alone in untreated mCRC suggested an improvement of response rate, PFS and OS with the combined treatment [7]. Then, the large randomized phase III study reported by Hurwitz et al. [6] demonstrated that the addition of bevacizumab to irinotecan and bolus 5FU/LV (IFL regimen) in first-line treatment was associated with a significant prolongation of PFS (6.2 months versus 10.6 months) and OS (15.6 months versus 20.3 months), as a better response rate (34.8% versus 44.8%). The results of that pivotal study led to the registration of bevacizumab in the fist-line setting in the United States and in Europe. The association of bevacizumab to 5FU/LV was also shown to provide a statistically significant and clinically relevant benefit to patients with previously untreated mCRC [6-8,32]. More recent studies showed that bevacizumab with FOLFIRI or FOLFOX4 regimen was superior to the chemotherapy alone in this setting [9,33]. In second-line, the ECOG E3200 phase III trial showed that bevacizumab improved response rate, PFS and OS when added to the FOLFOX4 regimen in patients pre-treated with a fluoropyrimidine and irinotecan [10]. Bevacizumab in combination with 5FU/LV plus irinotecan or oxaliplatin is therefore proved to be beneficial in first and 2^nd^-line setting. In the absence of cross-over after progression in the chemotherapy arm of the studies evaluating the addition of bevacizumab to a combined chemotherapy [6,10], the effect of the antiangiogenic agent associated with a bichemotherapy after the 2^nd^-line of treatment is not known. However, it was associated with a poor response rate (1%) in combination with a bolus regimen of 5FU/LV in chemotherapy-refractory mCRC [11]. In this large multicenter phase II study, PFS and OS were 3.7 months and 9.1 months respectively. This result, adding to the lack of efficacy of bevacizumab in monotherapy[10] led us to test it in this setting in combination with the more active FOLFOX4 or FOLFIRI regimens. Bevacizumab was administrated at the dose of 5 mg/kg every 2 weeks as it was reported in first-line studies with 5FU/LV/irinotecan or oxaliplatin [6,9,33] and since the 10 mg/kg dose was not previously shown to be superior [7].

Our results are consistent with those of a recent small study conducted in 14 mCRC patients that progressed after oxaliplatin and irinotecan and for whom bevacizumab plus infusional 5FU/LV and irinotecan allowed a 28.5% response rate with 57% of stabilized patients [34]. Median PFS was 3.9 months and OS was 10.9 months.

In our study, bevacizumab was well tolerated, with only one interruption due to a reversible posterior leukoencephalopathy syndrome associated with hypertension [14]. The other adverse events were easily manageable. As concerns chemotherapy-induced toxicity, it was relatively low because most of the patients previously received the same regimen as that associated with bevacizumab. Therefore required dose modifications were already performed before starting bevacizumab therapy

## Conclusion

Taking together, these results from retrospective data suggest that bevacizumab combined with FOLFOX or FOLFIRI may have the possibility to be active in chemorefractory and selected mCRC patients who did not receive it previously.

## Competing interests

AL and MY: received honoraria from Roche France

PR: received research grant from Roche France

## Authors' contributions

AL, JBB, ES, CBG collected the clinical data. AL, ES, EM, EA, CL, JBB, FP, JNV, MY and PR treated and followed the patients included in the study. PR, EM, MY and AL were involved in the conception of the study. EM performed the statistical analysis. AL wrote the manuscript. ES, EM, PR and MY were involved in the interpretation of data and critically revised the manuscript. All the authors read and approved the final manuscript.

## Pre-publication history

The pre-publication history for this paper can be accessed here:

http://www.biomedcentral.com/1471-2407/9/347/prepub

## References

[B1] de Gramont A Figer A Seymour M Homerin M Hmissi A Cassidy J Boni C Cortes-Funes H Cervantes A Freyer G Leucovorin and fluorouracil with or without oxaliplatin as first-line treatment in advanced colorectal cancer J Clin Oncol 2000 18 16 2938 2947 10944126 10.1200/JCO.2000.18.16.2938

[B2] Douillard JY Cunningham D Roth AD Navarro M James RD Karasek P Jandik P Iveson T Carmichael J Alakl M Irinotecan combined with fluorouracil compared with fluorouracil alone as first-line treatment for metastatic colorectal cancer: a multicentre randomised trial Lancet 2000 355 9209 1041 1047 10.1016/S0140-6736(00)02034-1 10744089

[B3] Goldberg RM Sargent DJ Morton RF Fuchs CS Ramanathan RK Williamson SK Findlay BP Pitot HC Alberts SR A randomized controlled trial of fluorouracil plus leucovorin, irinotecan, and oxaliplatin combinations in patients with previously untreated metastatic colorectal cancer J Clin Oncol 2004 22 1 23 30 10.1200/JCO.2004.09.046 14665611

[B4] Saltz LB Cox JV Blanke C Rosen LS Fehrenbacher L Moore MJ Maroun JA Ackland SP Locker PK Pirotta N Irinotecan plus fluorouracil and leucovorin for metastatic colorectal cancer. Irinotecan Study Group N Engl J Med 2000 343 13 905 914 10.1056/NEJM200009283431302 11006366

[B5] Ferrara N Molecular and biological properties of vascular endothelial growth factor J Mol Med 1999 77 7 527 543 10.1007/s001099900019 10494799

[B6] Hurwitz H Fehrenbacher L Novotny W Cartwright T Hainsworth J Heim W Berlin J Baron A Griffing S Holmgren E Bevacizumab plus irinotecan, fluorouracil, and leucovorin for metastatic colorectal cancer N Engl J Med 2004 350 23 2335 2342 10.1056/NEJMoa032691 15175435

[B7] Kabbinavar F Hurwitz HI Fehrenbacher L Meropol NJ Novotny WF Lieberman G Griffing S Bergsland E Phase II, randomized trial comparing bevacizumab plus fluorouracil (FU)/leucovorin (LV) with FU/LV alone in patients with metastatic colorectal cancer J Clin Oncol 2003 21 1 60 65 10.1200/JCO.2003.10.066 12506171

[B8] Kabbinavar FF Schulz J McCleod M Patel T Hamm JT Hecht JR Mass R Perrou B Nelson B Novotny WF Addition of bevacizumab to bolus fluorouracil and leucovorin in first-line metastatic colorectal cancer: results of a randomized phase II trial J Clin Oncol 2005 23 16 3697 3705 10.1200/JCO.2005.05.112 15738537

[B9] Saltz LB Clarke S Diaz-Rubio E Scheithauer W Figer A Wong R Koski S Lichinitser M Yang TS Rivera F Bevacizumab in combination with oxaliplatin-based chemotherapy as first-line therapy in metastatic colorectal cancer: a randomized phase III study J Clin Oncol 2008 26 12 2013 2019 10.1200/JCO.2007.14.9930 18421054

[B10] Giantonio BJ Catalano PJ Meropol NJ O'Dwyer PJ Mitchell EP Alberts SR Schwartz MA Benson AB 3rd Bevacizumab in combination with oxaliplatin, fluorouracil, and leucovorin (FOLFOX4) for previously treated metastatic colorectal cancer: results from the Eastern Cooperative Oncology Group Study E3200 J Clin Oncol 2007 25 12 1539 1544 10.1200/JCO.2006.09.6305 17442997

[B11] Chen HX Mooney M Boron M Vena D Mosby K Grochow L Jaffe C Rubinstein L Zwiebel J Kaplan RS Phase II multicenter trial of bevacizumab plus fluorouracil and leucovorin in patients with advanced refractory colorectal cancer: an NCI Treatment Referral Center Trial TRC-0301 J Clin Oncol 2006 24 21 3354 3360 10.1200/JCO.2005.05.1573 16849749

[B12] Therasse P Arbuck SG Eisenhauer EA Wanders J Kaplan RS Rubinstein L Verweij J Van Glabbeke M van Oosterom AT Christian MC New guidelines to evaluate the response to treatment in solid tumors. European Organization for Research and Treatment of Cancer, National Cancer Institute of the United States, National Cancer Institute of Canada J Natl Cancer Inst 2000 92 3 205 216 10.1093/jnci/92.3.205 10655437

[B13] Common Toxicity Criteria (CTC) v2.0 http://cancer.gov/

[B14] El Maalouf G Mitry E Lacout A Lievre A Rougier P Isolated brainstem involvement in posterior reversible leukoencephalopathy induced by bevacizumab J Neurol 2008 255 2 295 296 10.1007/s00415-008-0692-2 18283405

[B15] Chong G Dickson JL Cunningham D Norman AR Rao S Hill ME Price TJ Oates J Tebbutt N Capecitabine and mitomycin C as third-line therapy for patients with metastatic colorectal cancer resistant to fluorouracil and irinotecan Br J Cancer 2005 93 5 510 514 10.1038/sj.bjc.6602733 16091760 PMC2361607

[B16] Lim DH Park YS Park BB Ji SH Lee J Park KW Kang JH Lee SH Park JO Kim K Mitomycin-C and capecitabine as third-line chemotherapy in patients with advanced colorectal cancer: a phase II study Cancer Chemother Pharmacol 2005 56 1 10 14 10.1007/s00280-004-0963-2 15782313

[B17] Rao S Cunningham D Price T Hill ME Ross PJ Tebbutt N Norman AR Oates J Shellito P Phase II study of capecitabine and mitomycin C as first-line treatment in patients with advanced colorectal cancer Br J Cancer 2004 91 5 839 843 15266319 10.1038/sj.bjc.6602039 PMC2409883

[B18] Rimassa L Gullo G Carnaghi C Abbadessa G Zuradelli M Tronconi MC Pressiani T Santoro A Chemotherapy with mitomycin C and capecitabine in patients with advanced colorectal cancer pretreated with irinotecan and oxaliplatin Tumori 2006 92 4 285 289 17036517 10.1177/030089160609200404

[B19] Cunningham D Humblet Y Siena S Khayat D Bleiberg H Santoro A Bets D Mueser M Harstrick A Verslype C Cetuximab monotherapy and cetuximab plus irinotecan in irinotecan-refractory metastatic colorectal cancer N Engl J Med 2004 351 4 337 345 10.1056/NEJMoa033025 15269313

[B20] Jonker DJ O'Callaghan CJ Karapetis CS Zalcberg JR Tu D Au HJ Berry SR Krahn M Price T Simes RJ Cetuximab for the treatment of colorectal cancer N Engl J Med 2007 357 20 2040 2048 10.1056/NEJMoa071834 18003960

[B21] Van Cutsem E Peeters M Siena S Humblet Y Hendlisz A Neyns B Canon JL Van Laethem JL Maurel J Richardson G Open-label phase III trial of panitumumab plus best supportive care compared with best supportive care alone in patients with chemotherapy-refractory metastatic colorectal cancer J Clin Oncol 2007 25 13 1658 1664 10.1200/JCO.2006.08.1620 17470858

[B22] Amado RG Wolf M Peeters M Van Cutsem E Siena S Freeman DJ Juan T Sikorski R Suggs S Radinsky R Wild-type KRAS is required for panitumumab efficacy in patients with metastatic colorectal cancer J Clin Oncol 2008 26 10 1626 1634 10.1200/JCO.2007.14.7116 18316791

[B23] Benvenuti S Sartore-Bianchi A Di Nicolantonio F Zanon C Moroni M Veronese S Siena S Bardelli A Oncogenic activation of the RAS/RAF signaling pathway impairs the response of metastatic colorectal cancers to anti-epidermal growth factor receptor antibody therapies Cancer Res 2007 67 6 2643 2648 10.1158/0008-5472.CAN-06-4158 17363584

[B24] De Roock W Piessevaux H De Schutter J Janssens M De Hertogh G Personeni N Biesmans B Van Laethem JL Peeters M Humblet Y KRAS wild-type state predicts survival and is associated to early radiological response in metastatic colorectal cancer treated with cetuximab Ann Oncol 2008 19 3 508 515 10.1093/annonc/mdm496 17998284

[B25] Di Fiore F Blanchard F Charbonnier F Le Pessot F Lamy A Galais MP Bastit L Killian A Sesboue R Tuech JJ Clinical relevance of KRAS mutation detection in metastatic colorectal cancer treated by Cetuximab plus chemotherapy Br J Cancer 2007 96 8 1166 1169 10.1038/sj.bjc.6603685 17375050 PMC2360149

[B26] Lievre A Bachet JB Boige V Cayre A Le Corre D Buc E Ychou M Bouche O Landi B Louvet C KRAS mutations as an independent prognostic factor in patients with advanced colorectal cancer treated with cetuximab J Clin Oncol 2008 26 3 374 379 10.1200/JCO.2007.12.5906 18202412

[B27] Padera TP Stoll BR Tooredman JB Capen D di Tomaso E Jain RK Pathology: cancer cells compress intratumour vessels Nature 2004 427 6976 695 10.1038/427695a 14973470

[B28] Jain RK Normalization of tumor vasculature: an emerging concept in antiangiogenic therapy Science 2005 307 5706 58 62 10.1126/science.1104819 15637262

[B29] Willett CG Boucher Y di Tomaso E Duda DG Munn LL Tong RT Chung DC Sahani DV Kalva SP Kozin SV Direct evidence that the VEGF-specific antibody bevacizumab has antivascular effects in human rectal cancer Nat Med 2004 10 2 145 147 10.1038/nm988 14745444 PMC2693485

[B30] Hecht JR Mitchell E Chidiac T Scroggin C Hagenstad C Spigel D Marshall J Cohn A McCollum D Stella P A randomized phase IIIB trial of chemotherapy, bevacizumab, and panitumumab compared with chemotherapy and bevacizumab alone for metastatic colorectal cancer J Clin Oncol 2009 27 5 672 680 10.1200/JCO.2008.19.8135 19114685

[B31] Tol J Koopman M Cats A Rodenburg CJ Creemers GJ Schrama JG Erdkamp FL Vos AH van Groeningen CJ Sinnige HA Chemotherapy, bevacizumab, and cetuximab in metastatic colorectal cancer N Engl J Med 2009 360 6 563 572 10.1056/NEJMoa0808268 19196673

[B32] Kabbinavar FF Hambleton J Mass RD Hurwitz HI Bergsland E Sarkar S Combined analysis of efficacy: the addition of bevacizumab to fluorouracil/leucovorin improves survival for patients with metastatic colorectal cancer J Clin Oncol 2005 23 16 3706 3712 10.1200/JCO.2005.00.232 15867200

[B33] Fuchs CS Marshall J Mitchell E Wierzbicki R Ganju V Jeffery M Schulz J Richards D Soufi-Mahjoubi R Wang B Randomized, controlled trial of irinotecan plus infusional, bolus, or oral fluoropyrimidines in first-line treatment of metastatic colorectal cancer: results from the BICC-C Study J Clin Oncol 2007 25 30 4779 4786 10.1200/JCO.2007.11.3357 17947725

[B34] Kwon HC Oh SY Lee S Kim SH Kim HJ Bevacizumab plus infusional 5-fluorouracil, leucovorin and irinotecan for advanced colorectal cancer that progressed after oxaliplatin and irinotecan chemotherapy: a pilot study World J Gastroenterol 2007 13 46 6231 6235 10.3748/wjg.13.6231 18069765 PMC4171235

